# Iron-streptomycin derived catalyst for efficient oxygen reduction reaction in ceramic microbial fuel cells operating with urine

**DOI:** 10.1016/j.jpowsour.2019.03.052

**Published:** 2019-06-15

**Authors:** Maria Jose Salar Garcia, Carlo Santoro, Mounika Kodali, Alexey Serov, Kateryna Artyushkova, Plamen Atanassov, Ioannis Ieropoulos

**Affiliations:** a Bristol BioEnergy Centre, Bristol Robotics Laboratory, T-Block, UWE, Coldharbour Lane, Bristol BS16 1QY, UK; b Department of Chemical and Biological Engineering, Center for Micro-Engineered Materials (CMEM), Advanced Materials Lab, 1001 University Blvd. SE Suite 103, MSC 04 2790, Albuquerque, NM, 87131, University of New Mexico, USA; c Pajarito Powder, LLC, 3600 Osuna Rd NE Ste 309, Albuquerque, NM, 87109, USA

**Keywords:** Iron-based catalyst, Oxygen reduction reaction, Microbial fuel cells, Bioenergy

## Abstract

In recent years, the microbial fuel cell (MFC) technology has drawn the attention of the scientific community due to its ability to produce clean energy and treat different types of waste at the same time. Often, expensive catalysts are required to facilitate the oxygen reduction reaction (ORR) and this hinders their large-scale commercialisation. In this work, a novel iron-based catalyst (Fe-STR) synthesised from iron salt and streptomycin as a nitrogen-rich organic precursor was chemically, morphologically and electrochemically studied. The kinetics of Fe-STR with and without being doped with carbon nanotubes (CNT) was initially screened through rotating disk electrode (RDE) analysis. Then, the catalysts were integrated into air-breathing cathodes and placed into ceramic-type MFCs continuously fed with human urine. The half-wave potential showed the following trend Fe-STR > Fe-STR-CNT ≫ AC, indicating better kinetics towards ORR in the case of Fe-STR. In terms of MFC performance, the results showed that cathodes containing Fe-based catalyst outperformed AC-based cathodes after 3 months of operation. The long-term test reported that Fe-STR-based cathodes allow MFCs to reach a stable power output of 104.5 ± 0.0 μW cm^−2^, 74% higher than AC-based cathodes (60.4 ± 3.9 μW cm^−2^). To the best of the Authors' knowledge, this power performance is the highest recorded from ceramic-type MFCs fed with human urine.

## Highlights

• Novel iron-streptomycin derived catalyst for ORR in MFCs fed with urine.
• The half-wave potential results of Fe-STR show a better kinetic towards ORR.
• Fe-STR-based cathodes allow MFCs to reach a power output 74% higher than AC.
• The maximum power density output was 104.5 ± 0.0 μW cm^−2^ by using Fe-STR-cathodes.
• MFC performance was stable for 3 months working with Fe-STR-based cathodes.

## 1 Introduction

The Microbial fuel cell (MFC) is a promising biotechnology with the dual function of treating organic waste and generating electricity [1,2]. This bio-electrochemical system is supported by anode-respiring bacteria that break down organics within the liquid electrolyte releasing electrons to the solid electrode (anode) [3,4]. Electrons move through the external circuit generating a current flow that can be harvested. The redox reaction occurring within MFCs is completed at the cathode with the reduction of an oxidant.

A range of diverse organic waste has been successfully utilised as anodic fuel for MFCs [5,6]. In parallel, several elements and compounds, including nitrate [7], sulfate [8], oxygen [9], ferricyanide [10], permanganate [11] and other metal ions (e.g., Fe [12], Cu [13], Cr [14], etc.), have been investigated at the cathode half-cell as oxidising agents. Amongst them, oxygen is the best due to its intrinsic characteristics such as high reduction potential, low weight, and no cost.

In fuel cells operating at any pH values, the oxygen reduction reaction (ORR) is sluggish and therefore the limiting factor of the overall redox reaction of the system [15,16]. ORR can pursue two different routes depending on the supporting electrolyte that can be acidic or alkaline [17,18]. MFCs operate at near-neutral pH, and therefore it is not clear which pathway is followed. Alkalisation of the cathode has been reported, therefore leading to the conclusion that the alkaline pathway is preferred [[19], [20], [21], [22]].

To accelerate the sluggish cathodic reaction, a catalyst is always integrated within the cathode structure. Biological catalysts such as enzymes and bacteria have been investigated with some important drawbacks [23,24]. Whereas enzymes are very active in circumneutral pHs, the turn over frequency (TOF) despite being high, depends on the amount of enzymes trapped [25,26]. Also, enzymes are expensive and tend to denature rapidly, a process which is substantially accelerated in the presence of contaminants as recently shown [27,28]. The effectiveness of bacterial-based biocathodes is still under debate, and the electron transfer mechanisms are still discussed [23].

Inorganic materials seem to be more suitable as cathode catalysts in MFCs [[29], [30], [31], [32]]. Three types of inorganic catalysts have been used so far; namely, i) platinum group metals (PGMs), ii) metal-free carbonaceous-based materials and iii) platinum group metal-free materials [[29], [30], [31], [32]]. Additionally, ORR can follow diverse pathways during the reduction process following a 2e^−^, 2x2e^−^ and a direct 4e^−^ transfer mechanism. Metal-free carbonaceous catalysts follow a 2e^−^ transfer mechanism whereas platinum group metal-free (PGM-free) catalysts tend to follow a 2e^−^ or 2x2e^−^ transfer depending on the metal or atomic dispersion in a nanoparticle. Finally, PGM catalysts tend to follow a direct 4e^−^ transfer mechanism at high and low pHs.

PGM materials are the most utilised catalysts in MFCs [31]. These materials are quite active along the pH spectra, but due to their high cost and ease of poisoning in the presence of anions and pollutants, their utilisation in large scale is infeasible and illogical [33,34]. The low durability of PGM catalysts in MFCs was previously identified and studied [35,36].

An interesting option to replace costly PGM catalysts is represented by carbonaceous materials [37,38]. These materials are also known as metal-free catalysts despite nanoparticles or traces of metals can be found due to either the presence of impurities within the raw materials or during the preparation process. Different alternatives were adopted as carbonaceous materials such as carbon black [39], activated carbon [[40], [41], [42], [43], [44]], carbon nanotubes [45,46], carbon nanofibers [47], graphene [[48], [49], [50]], aerogel [51] and others [4,37,38]. Some of these materials can be found commercially at low cost whilst others can be easily synthesised at a relatively small price. On the contrary, other materials are quite complicated to manufacture and therefore expensive. The performance achieved by carbonaceous-based catalysts is quite promising despite the activation overpotentials remaining quite high and quantified in 400–500 mV [4,37,38].

PGM-free catalysts exhibit higher performance than carbon-based materials and seem to be a realistic alternative to PGM. PGM-free catalysts are generally based on transition metals such as Fe [[52], [53], [54], [55], [56], [57], [58], [59], [60]], Co [[61], [62], [63], [64]], Ni [65,66] or Mn [[67], [68], [69], [70]]. Two main categories have been explored, one based on the synthesis through high temperature treatment of a metal salt with a nitrogen-rich organic precursor [[71], [72], [73], [74]] and the second one based on the use of organic molecules (e.g., porphyrins, phthalocyanines, etc.) in which the transition metal is integrated [70,[75], [76], [77]]. Both categories of PGM-free catalysts show high performance levels towards the reduction of oxygen as well as in operating MFCs, and long-term stability [[78], [79], [80]]. PGM-free catalysts based on Fe as the active catalytic center show higher performance in rotating ring-disk electrode (RRDE) [81] and in operating MFCs compared to Co, Mn and Ni [82]. Co was the second best followed by Mn and Ni [82]. According to these results, the utilisation of Fe-based PGM-free catalysts is recommended.

Moreover, Fe is more environmentally friendly and widely available compared to the other transition metals mentioned above. On the other hand, the existing literature also reports that the addition of Fe-based catalysts significantly increases the performance of the ORR [83]. It should also be mentioned that Fe—N—C ORR catalysts are available (e.g. Pajarito Powder, LLC [https://pajaritopowder.com/]).

In this work, an iron-based catalyst synthesised from iron salt and streptomycin as a nitrogen-rich organic precursor was chemically, morphologically and electrochemically studied. The kinetics of Fe-streptomycin was initially screened through RDE analysis as well as the catalyst doped with carbon nanotubes. Then both catalysts were independently integrated into air-breathing cathodes and placed into a microbial fuel cell with a ceramic separator continuously fed with urine. The performance of the MFCs was monitored over 90 days and data are reported herewith.

## 2 Materials and method

### 2.1 Catalyst preparation

The Fe-based catalysts were synthesised using the Sacrificial Support Method (SSM) as previously described [55]. To increase the material pores sizes, two different types of silica, one with a medium surface area called LM150 (150 m^2^ g^−1^) and one with a low surface area called OX50 (45 m^2^ g^−1^), were used as templating agents obtaining an overall surface area of ∼600 m^2^ g^−1^. Firstly, a colloidal solution of the two silica was made, and then streptomycin was added. Streptomycin is a low-cost nitrogen-rich organic precursor. In the case of the catalyst with the addition of carbon nanotubes, CNTs (CheapTubes, LLC, USA) were added after adding streptomycin. After mixing the solution vigorously, FeNO_3_*9H_2_O (Sigma Aldrich), was added as a metal salt. The mixture was moved to a stirring hot plate in which the temperature was increased up to 45 °C allowing the water evaporation from the solution. The temperature was further increased up to 85 °C to dry the viscous solution. The dried powder was then ground in a pestle and mortar to a fine powder, which was placed into a ceramic boat where a high-temperature treatment is applied (pyrolysis). Particularly, the temperature was increased up to 975 °C (10 °C min^−1^ ramp) and maintained for 45 min, while within the quartz tube inert atmosphere of ultra high pure (UHP) nitrogen was kept at a flow rate of 100 ml min^−1^. The silica template was removed through the utilisation of hydrofluoric acid (HF) with a 25%wt concentration that was in contact with the catalyst for 24 h. After the etching procedure, the catalyst was washed using deionised water several times until the pH of the solution was neutral. The obtained powder was then dried at 80 °C overnight. The obtained catalysts were abbreviated as Fe-STR and Fe-STR-CNT.

### 2.2 Surface chemistry and morphology

High-resolution X-Ray photoelectron spectroscopy (XPS) was performed using Kratos Ultra DLD XPS spectrometer. Samples were placed onto a conductive carbon tape. Three areas per sample were analysed, and the average, as well as the standard deviation, are reported for each sample. No charge neutralisation was done. Monochromatic Al K alpha source operating at 225 kW was employed. High-resolution spectra were acquired at a pass energy of 20 eV. CasaXPS was used to process the data. The elemental composition was obtained by using relative sensitivity factors provided by the manufacturer. Graphitic carbon was fitted using asymmetric peak in C 1s spectra, while the rest of the peaks had symmetric 70/30 Gaussian/Lorentzian shape. SEM images were obtained by using Hitachi S-5200 Nano scanning electron microscope (an accelerating voltage of 10 keV) and JEOL JEM-2010.

### 2.3 Rotating disk electrode (RDE)

The rotating disk electrode (RDE) technique was used for studying the kinetics of the catalysts. The instrument used was an RDE (Pine Research, USA) with a glassy carbon electrode. Inks were formulated from the catalyst investigated (AC, Fe-STR, and Fe-STR-CNT). The three inks were composed of 850 μL of IPA:H_2_O (ratio 1 to 4) and 150 μL of 0.5 wt% Nafion with 5 mg of each catalyst. Each ink was ultra-sonicated and shaken for 4 and 3 min respectively, each procedure being repeated three times. Drop casting technique was used for depositing the ink on the glassy carbon electrode that was then dried naturally before running the experiment. The catalyst loading investigated was 200 μg cm^−2^.

The tests were conducted in potassium phosphate buffer (K-PB) 0.1M at pH = 9 with 0.1 M KCl to simulate the pH of zhuman urine. The buffer was purged with pure oxygen before running the tests. Linear sweep voltammetry (LSVs) was run using a Pine potentiostat in a three-electrode configuration with the rotating glassy carbon as a working electrode, Ag/AgCl 3M KCl as a reference electrode and a graphite rod as the counter electrode. LSVs were performed from +0.4 V vs (Ag/AgCl) to −0.9 V vs (Ag/AgCl) at a scan rate of 5 mV s^−1^. Disk current, named as I_disk_, was reported and the onset potential (E_onset_), a half-wave potential (E_1/2_) and limiting current density (i_lim_) were identified and compared. The discussion on E_onset_, E_1/2_ and i_lim_ was done for the rotating speed of 1600 rpm.

### 2.4 Air-breathing cathode preparation

The different catalysts synthesised in this work were then integrated into air-breathing cathodes as described before [55]. Particularly, activated carbon (AC, Norit SX Ultra), carbon black (CB, Alfa Aesar, acetylene black 50 wt% compressed) and polytetrafluoroethylene (PTFE, 60 wt% emulsion Sigma Aldrich) were blended in a weight ratio of 7:1:2, respectively. The black mixture was then weighted and pressed over a stainless steel mesh SS316 that worked as a current collector. The AC cathode, also used as a control, had a loading of AC-CB-PTFE of 45 mg cm^−2^. Fe-STR and Fe-STR-CNT were prepared similarly, but within the mixture of AC-CB-PTFE, the catalyst was added and mixed manually making a homogeneous blend. Fe-STR and Fe-STR-CNT had a loading of 45 mg cm^−2^ where 40 mg cm^−2^ was composed by AC-CB-PTFE, and 5 mg cm^−2^ was loading of the Fe—N—C-based catalyst. It was shown previously that different AC loading does not affect the power output [84].

### 2.5 Anode electrode and microbial fuel cell

Anodes were made of carbon veil (30 g m^−2^, PRF composites, Dorset. the UK). The geometric anode area selected was 102.25 cm^2,^ and it was folded and placed in the anodic chamber with an empty volume of 12.5 mL. Flat terracotta membranes (3.5 × 3.5 cm^2^) were handmade by kilning square pieces of terracotta clay for 3 min at 1070 °C (time ramp 7 h) with a final thickness of 1.6 mm. The MFC set-up consisted of a cubical assembly made of acrylic material. The active part of the cathode was faced the terracotta whereas the stainless steel was exposed to air.

### 2.6 MFC operation

MFCs were initially fed with a solution of sludge and human urine (1:1 v/v) in batch mode. The MFCs were subject to four cycles of 24 h each, in which the solution was fully replenished. The feeding was then switched to urine in a continuous flow of 0.06 mL min^−1^. During the anode maturing stage (7 days), the external loading of the system was varying from 1000 Ω to 500 Ω to facilitate biofilm growth. These values were determined from earlier work on this same type of MFC set-up. MFC voltage was continuously monitored by using a 16-channel ADC-24 Picolog recorder data logger (Pico Technology Ltd, Cambridgeshire, UK) during 90 days. Each cathode prepared was assessed in duplicate.

### 2.7 Electrochemical measurements

The polarisation of MFCs using the different cathodes elaborated was performed using a potentiostat (AUTOLAB III/FRA2, Metrohm, The Netherlands) by linear sweep voltammetry (LSV) from open circuit voltage (OCV) to 0.02 mV at a scan rate of 0.25 mV s^−1^. The MFCs were left in OCV for at least 2 h before performing the measurements to allow the stabilisation of the OCV. The two-electrode technique was used with the anode connected to the counter electrode, a cathode connected to the working electrode and reference channel short-circuited with the counter electrode channel. Polarisation curves were obtained by plotting the cell voltage *versus* current (V *vs.* I) whereas power curves were obtained by plotting power *versus* current (P *vs.* I). Power was obtained by multiplying voltage and current. The obtained power was also plotted as power density in function of the geometric area of the cathode that was 12.25 cm^2^.

LSV was also employed for characterising the anode and cathode separately in a three-electrode configuration. Particularly, the anode polarisation curve was done using the anode as a working electrode, a cathode as a counter and Ag/AgCl 3M KCl as a reference electrode. The anode LSV was run between anode open circuit potential (OCP) and −0.1 mV *vs.* Ag/AgCl. Three-electrode configuration was also used for characterising the cathode. Particularly, the cathode was connected to the working electrode channel, the anode worked as a counter electrode and Ag/AgCl 3M KCl operated as a reference electrode. The cathode LSV was run between cathode OCP and −0.3 mV *vs.* Ag/AgCl. Both anode and cathode LSVs were run at a scan rate of 0.25 mV s^−1^.

## 3 Results and discussion

### 3.1 Surface morphology and surface chemistry

**Fig. 1:**
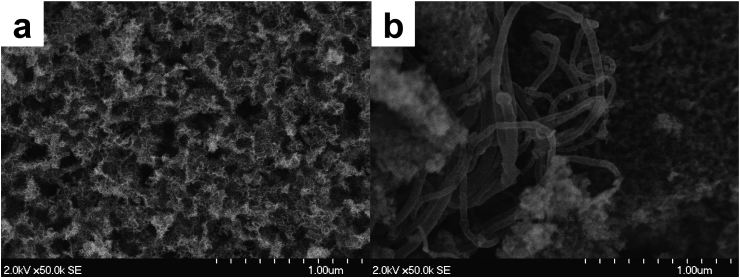
SEM image of Fe-STR (a) and (b) Fe-STR-CNT.

The morphology of the two novel catalysts investigated, Fe-STR and Fe-STR-CNT, was studied using SEM images (Fig. 1). For Fe-STR, a rough surface with two types of pore can be observed; the larger pores (left) after the removal of silica template and the smaller pores due to the gas-forming decomposition of the organic precursor molecules, shown on the right (Fig. 1a). Carbon nanotubes with a diameter between 80 and 100 nm can be clearly seen within the catalyst structure of Fe-STR-CNT (Fig. 1b).

Based on the surface analysis by XPS, the total nitrogen concentration in the samples varied between 3.1% (Fe-STR-CNT) and 4.1% (Fe-STR) with higher concentration in the CNT-free sample (Table 1). The incorporation of CNTs causes both a slight decrease in nitrogen and also an increase in oxygen concentration (from 4.8% to 7.6%) due to the presence of surface oxide groups on the surface of the CNTs. High-resolution C 1 spectra were fitted using multiple peaks with a major contribution from graphitic C=C (284.2 eV), aliphatic C—C (285 eV), C—N species (286 eV) and multiple peaks due to carbon oxides (287–291 eV). The catalyst with CNTs has a larger relative amount of graphitic carbon and smaller relative percent of aliphatic carbon in comparison with the CNT-free alternative. The same amount of carbon-oxygen species is detected in both samples suggesting that the increase of the amount of oxygen observed in the CNT-containing sample is not due to carbon surface oxides (Table 1).

**Table 1:** Elemental composition and relative composition from the deconvolution of N 1s spectra and C 1s spectra.

	C 1s %	N 1s %	O 1s %
Fe-STR	91.1 ± 0.2	4.1 ± 0.4	4.8 ± 0.2
Fe-STR-CNT	89.2 ± 1.9	3.1 ± 0.2	7.6 ± 0.7

	N	N	N_x_-Me	N—H	N_gr_/N^+^	N—H	NO	NO_2_—NO_3_
	imine	pyrid				bulk		
Fe-STR	3.9 ± 0.2	21.8 ± 1.6	12.2 ± 2.4	30.3 ± 0.3	17.4 ± 0.4	7.7 ± 0.0	2.0 ± 0.3	4.6 ± 0.2
Fe-STR-CNT	2.8 ± 1.0	17.3 ± 1.4	14.5 ± 1.0	29.1 ± 2.6	11.9 ± 2.6	4.8 ± 0.1	3.0 ± 1.5	16.8 ± 1.5

	C_gr_	C—C/C*	C—N	C_x_O_y_
Fe-STR	14.5 ± 2.8	44.1 ± 2.3	18.1 ± 0.2	23.3 ± 0.2
Fe-STR-CNT	26.2 ± 0.4	34.9 ± 4.9	16.0 ± 2.7	22.4 ± 0.8

It has been shown before that Fe-based catalysts prepared using SSM method are subject to a variety of defects and of major interest is the nitrogen- and iron-containing moieties [85]. It has also been reported that nitrogen (up to 10%) enhances the electrical conductivity of the catalysts [85] and helps to improve the electrocatalytic activity towards ORR [85]. Particularly, nitrogen pyridinic and nitrogen coordinated to metal were able to enhance the catalytic activity measured both in RDE and in air-breathing cathodes for MFCs [55].

**Fig. 2:**
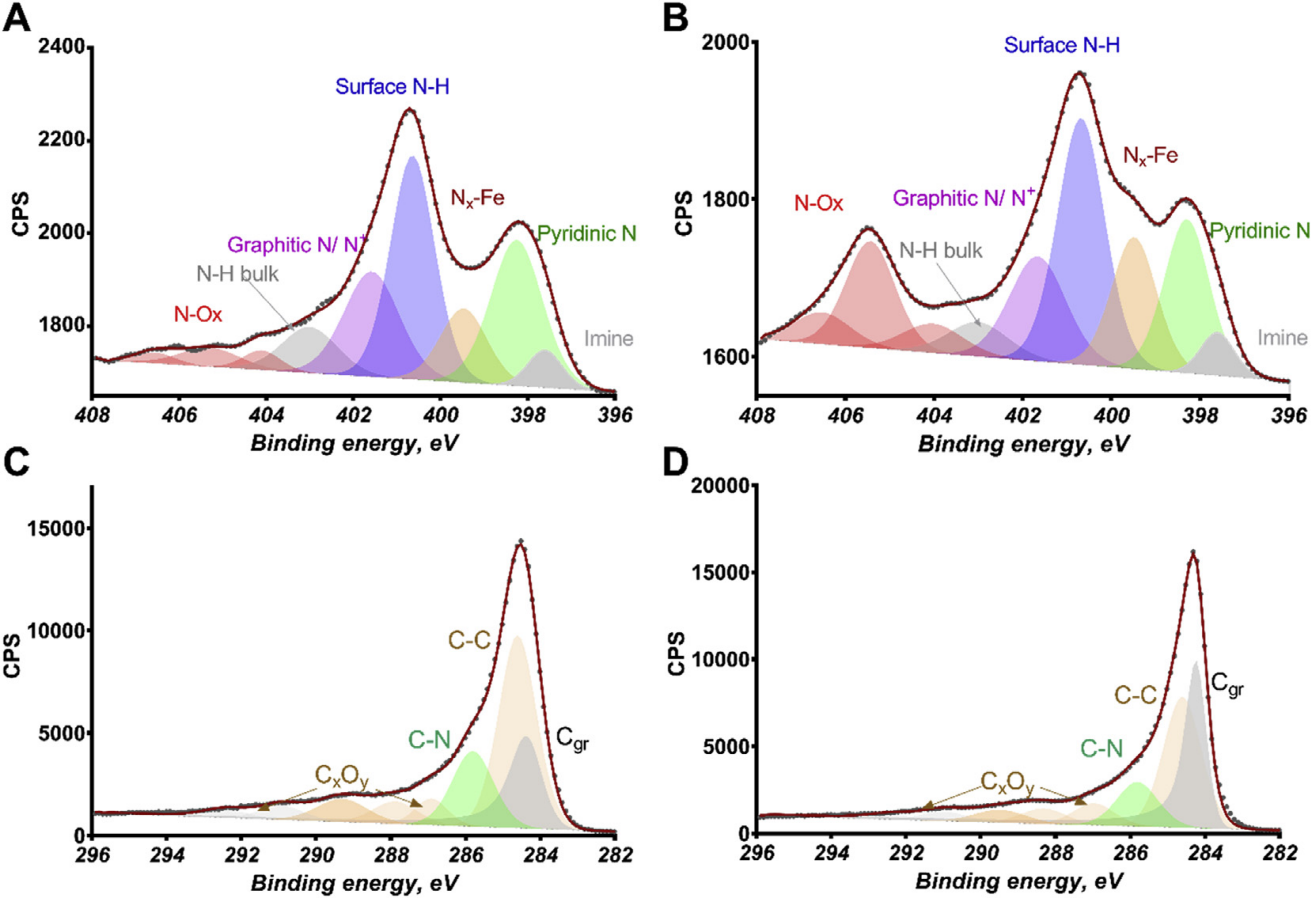
XPS. Spectra of the N 1s for Fe-STR (a) and Fe-STR-CNT (b) and spectra of the C1s for Fe-STR (c) and Fe-STR-CNT (d).

High-resolution N 1s spectra were fitted with multiple components according to a fit described in previous reports [85]. For consistency, the position and width of the peaks were adapted from analysis of materials from the same family (Fig. 2). Lowest binding energy component is due to imine N (397.5 eV). Peaks coming from pyridinic N (398.3 eV) and nitrogen coordinated to iron (399.4 eV) have been shown to be important metrics of high catalytic activity in ORR [85]. Pyridinic nitrogen had a relative percentage that varied between 17.3% (Fe-STR-CNT) and 21.8% (Fe-STR). Hydrogenated nitrogen contributes to two peaks: lower BE (401 eV) representing surface edge N—H sites incorporated in graphene edge terminated with carbon surface oxides and higher binding energy peak (403 eV) due to bulk N—H defect sites incorporated within graphene sheets terminated with C—H edge groups. Graphitic and protonated nitrogens are detected at 402 eV. Multiple types of N—O species are contributing to peaks above 403 eV. The greatest difference between CNT-free and CNT-containing catalyst is in contribution from nitrogen oxides. The highest amount of peaks belong to the NO_2_—NO_3_ species as observed for CNT-containing samples. This confirms that the larger amount of oxygen shows at the surface of CNT-containing material is caused by the presence of oxidised nitrogen species (Table 1).

### 3.2 RDE analysis

**Fig. 3:**
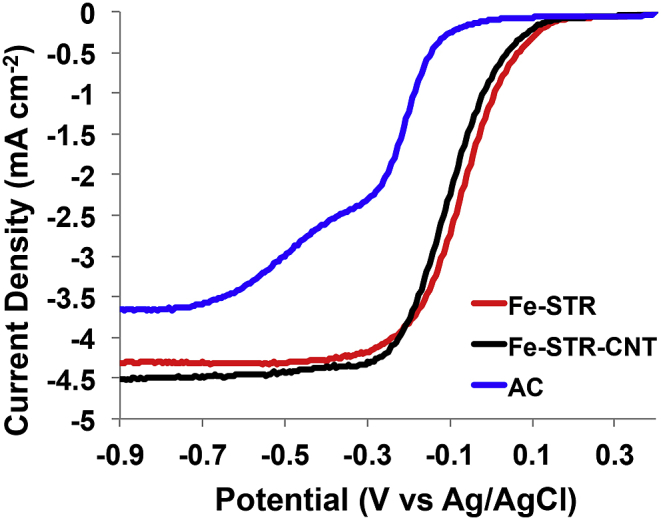
Disk current for Fe-STR, Fe-STR-CNT, and AC at a rotation of 1600 rpm in oxygen saturated electrolyte at pH = 9.

RDE analyses were carried out for identifying the electrocatalytic activity of the catalysts at pH = 9 in oxygen saturated electrolyte (Fig. 3). E_onset_ of Fe-based catalysts was higher compared to AC. Particularly, Fe-STR had an E_onset_ of 180.1 mV (vs Ag/AgCl) followed closely by Fe-STR-CNT (169.9 mV (vs Ag/AgCl)) and AC with the lowest E_onset_ (−14.9 mV (vs Ag/AgCl)). The kinetics of the reaction are often identified by the half-wave potential (E_1/2_). Fe-STR had slightly higher E_1/2_ compared to Fe-STR-CNT. In detail, the E_1/2_ for Fe-STR was −5 mV (vs Ag/AgCl) and for Fe-STR-CNT was −8 mV (vs Ag/AgCl). AC E_1/2_ is difficult to identify due to the shape of the curve, but it was certainly 200 mV lower than that observed in the case of Fe-based catalysts. This indicates that Fe-based catalysts had much higher electrocatalytic activity compared to AC. The same trend was observed previously, where Fe-based, Co-based, Mn-based and Ni-based atomically dispersed PGM-free catalysts outperformed bare AC [82]. Concerning the limiting current (i_lim_), Fe-STR-CNT had a value of 4.5 mA cm^−2^ followed by Fe-STR with 4.3 mA cm^−2^ and AC with 3.6 mA cm^−2^. It was previously reported that a strict relationship between the RDE data and the catalyst performance once integrated into the air-breathing cathode, in both clean media and operating MFC. It was also shown that RDE data could predict the performance of the catalyst once operating in MFCs [55].

### 3.3 MFC stability test

**Fig. 4:**
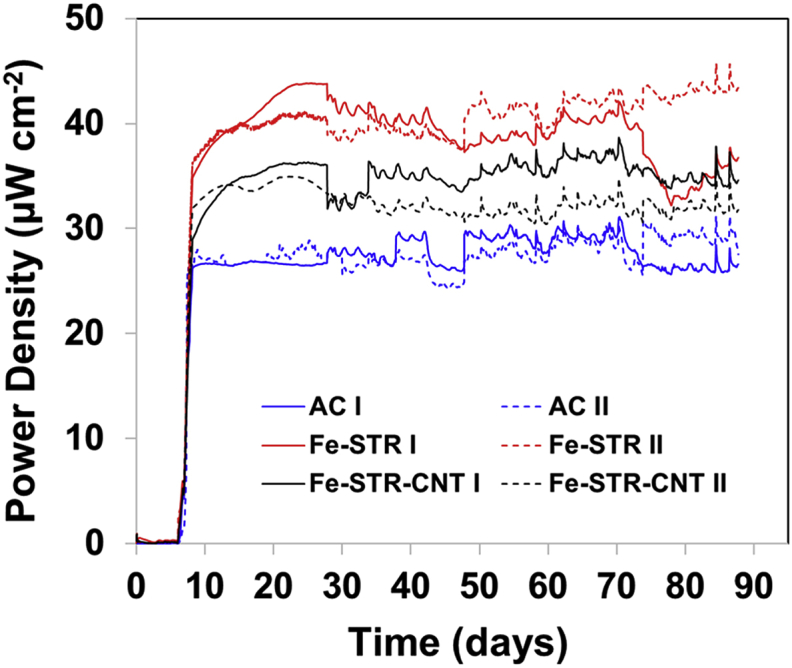
Power density trend of MFCs over 90 days of operating time.

Long-term stability of MFC is a key aspect to consider concerning their practical applications. In this work, after the inoculation phase (inoculum refreshed every day), the MFCs were run over a relatively long period of 3 months (90 days) under a constant external resistance of 500 Ω. This value was selected according to previous results not shown in this work but which allowed the MFCs to reach a stable electrochemical performance. Once the system was matured (after 7 days), the voltage remained stable for more than 60 days. Fig. 4 depicts the evolution of the power density over time for each material assessed in duplicate. During the operating time, once the steady state was reached (around day 20), Fe-STR-based cathodes reached a voltage of 41.2 ± 0.02 μW cm^−2^ whereas MFCs working with Fe-STR-CNT-based cathodes showed a lower voltage of 35.3 ± 0.01 μW cm^−2^. AC-CB-PTFE-based cathodes had the lowest voltage measured in this work (26.9 ± 0.01 μW cm^−2^). All materials tested exhibit good long-term stability, which would benefit the scale-up of MFC technology.

### 3.4 Electrochemical performance after one month and three months running

**Fig. 5:**
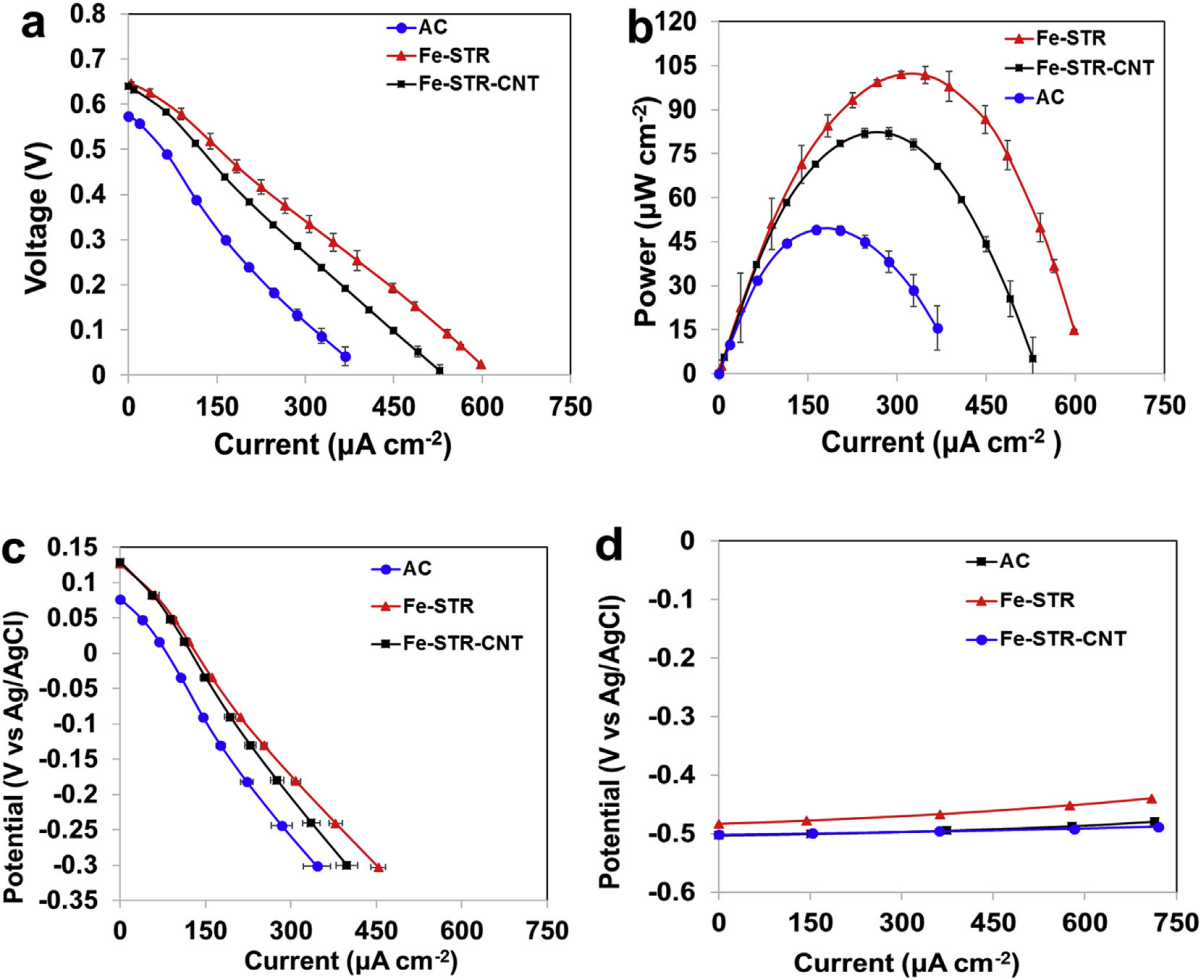
Polarisation curves (a), power curves (b), cathode polarisation (c) and anode polarisation (d) of the three different MFCs after one-month operation.

**Fig. 6:**
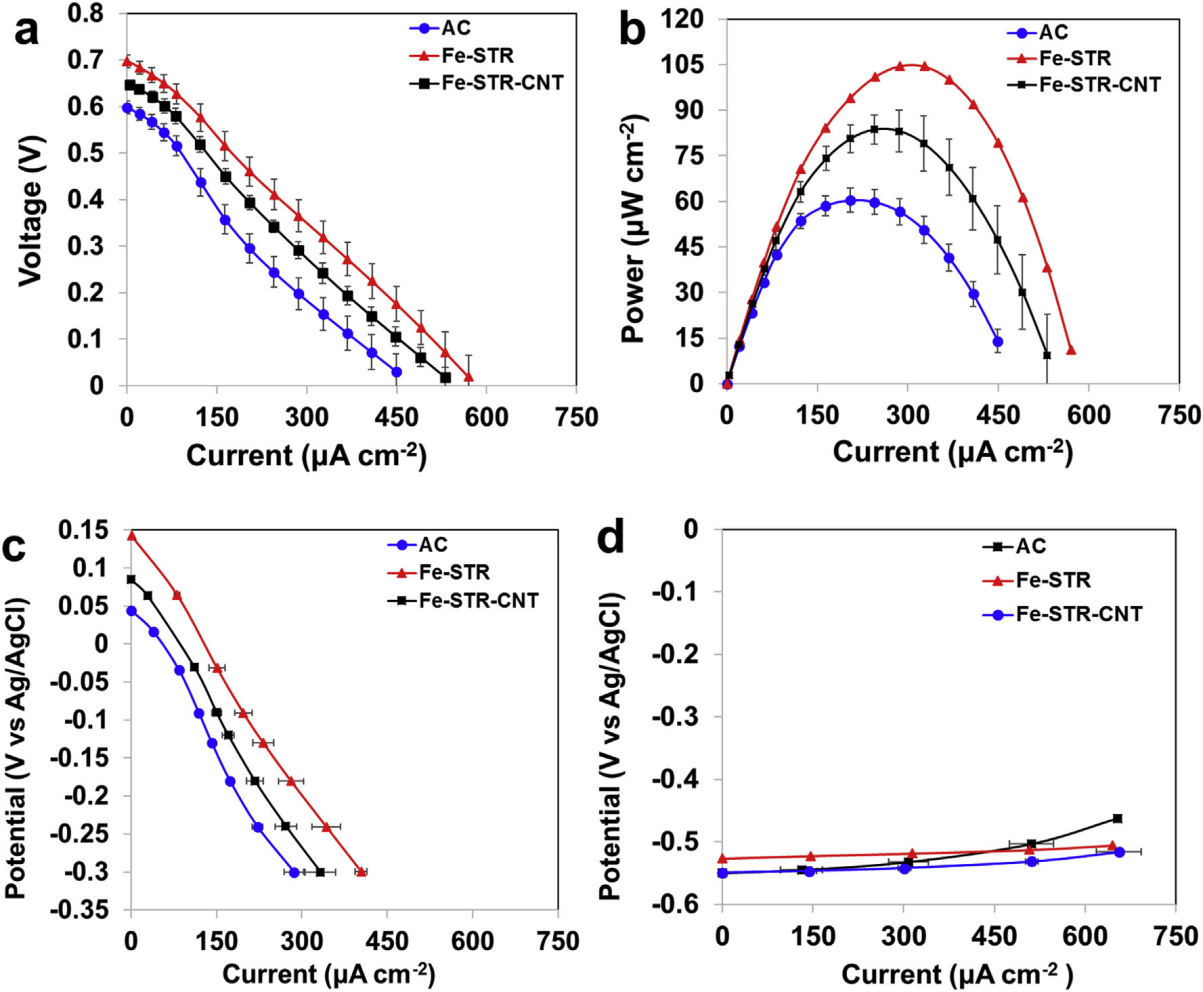
Polarisation curves (a), power curves (b), cathode polarisation (c) and anode polarisation (d) of the three different MFCs after three-month operation.

The performance of MFCs in terms of power output was evaluated, and the behaviour of both anodes and cathodes was analysed individually. Fig. 5 and Fig. 6 show the electrochemical performance of both the overall cell and the single electrodes after 1 month and 3 months of operating time, respectively. As can be observed, in both cases after 1 and 3 months, the open circuit voltage (OCV) of the MFC with Fe-STR and Fe-STR-CNT based cathodes was higher compared to the system with AC based cathode (Fig. 5.a and Fig. 6.a, respectively). Interestingly, the overall polarisation curves showed that MFCs with Fe-STR integrated air-breathing cathode exhibit higher electrocatalytic activity compared to Fe-STR-CNT. The performance of MFCs based on plain AC cathodes was lower than the other two cases. MFC with Fe-STR cathodes reached a maximum power output of 102.1 ± 0.9 μW cm^−2^ (1250.7 ± 10.7 μW). The power density remained stable showing 104.5 ± 0.0 μW cm^−2^ (1280.6 ± 120.0 μW) after three months of operation. The incorporation of CNT within the catalyst led to a decrease in the performance. The Fe-STR-CNT based cathode produced 81.9 ± 1.6 μW cm^−2^ (1003.8 ± 19.6 μW) after 1 month and 83.7 ± 4.81 μW cm^−2^ (1024.9 ± 59 μW) after 3 months. MFC with AC-based cathode exhibited the lowest performance. After 1 month of operation, the power density was 49.2 ± 0.5 μW cm^−2^ (602.9 ± 6.4 μW), and it increased by 20%–60.4 ± 3.9 μW cm^−2^ (739.5 ± 47.9 μW) after 3 months. Table 2 summarises the maximum power output reached by MFCs containing different cathodes synthesised. During the first month, the power outputs of Fe-STR-CNT and Fe-STR cathodes were 73% and 105% higher compared to AC-based cathodes, respectively. After 3 months of operation, the advantage of these catalysts compared to plain AC decreased to 39% and 74%, respectively. These improvements underline the benefits of including platinum-free Fe-based catalysts for higher MFC output performance.

**Table 2:** The peak of power curves after 1 month and three months.

	1 month		3 months	
	μW	μW cm^−2^	μW	μW cm^−2^
AC	602.9 ± 6.4	49.2 ± 0.5	739.5 ± 47.9	60.4 ± 3.9
Fe-STR	1250.7 ± 10.7	102.1 ± 0.9	1280.6 ± 120.0	104.5 ± 0.0
Fe-STR-CNT	1003.8 ± 19.6	81.9 ± 1.6	1024.9 ± 59	83.7 ± 4.8

The single electrode polarisations were also recorded. The cathode curves indicated that Fe-STR outperformed Fe-STR-CNT and AC–based system both after one (Fig. 5c) and three months of operation (Fig. 6c). The anode polarisation curves were similar for all MFCs after both one (Fig. 5d) and three months of the test (Fig. 6d). These results indicate that the variation in the cathode behaviour is the main factor responsible for the differences in the overall MFCs performance described above.

## 4 Discussion

Two novel catalysts were synthesised using a sacrificial support method (SSM) and were tested in RDE and MFC once integrated into the air-breathing cathode. The catalysts, Fe-STR and Fe-STR-CNT, were both synthesised using iron nitrate and streptomycin as precursors. CNTs were added during the synthesis process to make Fe-STR-CNT material. RDE data showed that Fe-based catalysts outperformed AC. Among the Fe-based materials tested, Fe-STR had slightly higher E_1/2_ than the catalyst with the addition of CNTs. It was shown previously that the surface chemistry of the catalyst is related to its catalytic activity with specific N moieties being responsible for enhancing ORR [85]. The amount of total nitrogen, as well as the relative amount of pyridinic nitrogen, is higher for Fe-STR-based cathode than for the other synthesised catalysts.

Moreover, Fe coordinated to N has been shown to be responsible for a direct 4e-transfer mechanism, leading to improvement of the ORR [85]. Even though the relative percentage of these active catalytic sites was lower in the Fe-STR catalyst compared to that in Fe-STR-CNT, taking into account higher overall N content in the Fe-STR, it has a larger absolute surface concentration of N_x_-Fe centres than Fe-STR-CNT. Nitrogen oxides were shown previously not to be beneficial for ORR [85] and in this case, the addition of CNT increased the percentage of NO, NO_2_—NO_3_ species significantly (Table 1). The highest E_1/2_ obtained in this work was 180.1 mV (vs. Ag/AgCl) that is comparable with other Fe-based catalysts tested in previous works with identical conditions [85].

After studying the catalyst kinetic in RDE, the catalysts were incorporated into the air-breathing cathodes and tested in MFCs. MFCs with a ceramic-based separator and air-breathing cathodes were run over three months of operating time. The MFCs were fed continuously with human urine for the entire essay. The work aimed at evaluating the potential improvement of MFC performance by the addition of low-cost iron-based catalysts based on atomically dispersed iron. The results showed high stability over three months in terms of both voltage over a fixed resistance and during polarisation curves. The peak power produced was stable during the three months of operating. The maximum power density produced was 102.1 ± 0.9 μW cm^−2^ (1250.7 ± 10.7 μW, for Fe-STR cathode) and 104.5 ± 0.0 μW cm^−2^ (1280.6 ± 104.5 μW, for the Fe-STR-CNT cathode) after 1 and 3 month, respectively. AC-based cathode, which was used as a control, was the worst performing with 49.2 ± 0.5 μW cm^−2^ (602.9 ± 6.4 μW) after 1 month and 60.4 ± 3.9 μW cm^−2^ (739.5 ± 47.9 μW) after 3 months of operation. The advantage of using Fe-STR was 105% (first month) and 73% (third month) compared to AC cathode and ≈20% compared to Fe-STR-CNT (both 1 and 3 months). These results show a trend similar to previously observed in the RDE analysis. RDE data, thus, can be reliably used to predict the performance of the catalysts once they are integrated into MFCs, in agreement with the previously reported analysis [55].

Importantly, this is the highest power density (normalised to cathode area) obtained thus far for an MFC fed with human urine. Previously, in an MFC with a cylindrical configuration, fed also with urine, a peak of power density below 80 μW cm^−2^ was obtained using Fe-based catalysts [86].

Existing literature reports values above 200 μW cm^−2^ but these data were obtained by using synthetic feedstock such as phosphate buffer [87] or phosphate buffer mixed with activated sludge [55] and acetate as carbon source. Moreover, a ceramic separator was used, and this certainly increases the ohmic resistance of the cathode and the overall MFC. The experiments here presented were also conducted at room temperature, and it is well known that the increase in temperature positively affects the electrochemical output [88].

In previous reports, a decrease in the cathode catalytic activity over time was presented. Zhang *et al*. showed a reduction in the performance of up to 40% over 12 months [80], whereas Pasternak *et al*. showed a 90.7% decrease in performance due to biofouling after 3 months of continuous operation [95]. Similarly, Rossi *et al*. showed a decrease in maximum power by 26% after 2 months of operation [87]. When Fe-based catalysts, Fe-Ricobendazole and Fe-Niclosamide, were tested in a single chamber membrane-less MFCs, the activity decreased by roughly 20% during one-month operation [36]. A more drastic decrease was noticed by Mecheri *et al*. in which the decrease in power generation of roughly 50%, detected after one-month of operation, then the performance stabilised in the additional month of the test [89]. A much lower decrease in performance is usually observed when AC is used as a cathode catalyst in single chamber membrane-less MFC [44]. Catalyst poisoning of organic/inorganic catalysts is the main factor responsible for the decrease in the cathode activity over time [90]. The poisoning of the Fe-based catalyst due to pollutants is not fully investigated and understood, but initial results showed that M-N—C catalysts are more resilient to poisoning compared to platinum catalysts [91]. Preliminary work showed that M-N—C are poisoned by sulfur and by ammonium ions, but their deactivation is much slower compared to that observed for platinum catalyst [92]. Organic fouling seems to be more likely responsible for consuming the oxygen before it reaches catalytic sites. The removal of biofouling was found to be beneficial for recovering the performance [80,95]. The possibility of removing the biofilm *in situ* was recently shown by using magnetic stirrer in the internal part of the cathode [93]. Besides, inorganic fouling caused by the precipitation of salts has been shown to affect the cathode performance negatively. Inorganic fouling forms precipitates on the catalyst sites increasing the mass transfer resistance or covering completely the catalytic sites that are unable to perform the reaction [94]. X-ray computed microtomography was recently used to identify the inorganic fouling of cathodes during long-term operation, and it was assumed to be the main cause of the degradation of the cathode performance [94].

Interestingly, in this work conducted in a continuous flow using human urine, the performance did not vary during 3 months of operating time for both Fe-STR and Fe-STR-CNT based cathodes. In the case of AC-based cathodes, their performance slightly increased over time. It must be noted that the urine utilised in this work was already hydrolysed simply by being stored in the laboratory collection tank for ~24 hours with pH measured above 9 and, therefore, it is assumed that a large part of the salts (e.g., struvite, hydroxyapatite, calcium phosphate, etc.) had already precipitated in the collection tank. These durability tests conducted with real organic waste are certainly encouraging for the wider implementation of these long-term performing catalysts.

## 5 Conclusions

Two novel Fe-based catalysts synthesised using sacrificial support method were tested in rotating disk electrode and in the cathode of ceramic-based microbial fuel cells fed with urine. Fe-STR and Fe-STR-CNT had higher electrochemical parameters (onset potential, half-wave potential and limiting current) compared to activated carbon (AC) used as a control. Moreover, Fe-STR had a slightly higher half-wave potential compared to Fe-STR-CNT indicating a better kinetic towards ORR. Both Fe-based catalysts outperformed AC after 3 months working as MFC cathode (up to 74% higher). The power density referred to the cathode area obtained by Fe-STR (104.5 ± 0.0 μW cm^−2^) was the highest ever recorded from an MFC fed with human urine. Stability tests showed that the MFCs exhibited stable performance over 3 months of study. All the results reported in this work support that the utilisation of low-cost Fe-based catalysts for boosting the performance output is certainly an important pathway to be pursued.
